# Fewer Frogs in Illinois: Organochlorines May Be to Blame

**Published:** 2005-03

**Authors:** Renée Twombly

To understand the worldwide decline in amphibian populations, many researchers are looking at the current use of industrial compounds that disrupt endocrine function and lead to impaired reproduction. But a group of researchers in Illinois thought a historic perspective might offer some additional clues **[*****EHP*** **113:261–265]**. What they found reveals a new possibility as to why the cricket frog, indigenous to the eastern half of the United States, has experienced a marked population decline in recent decades.

Endocrine systems evolved to yield exquisite sensitivity to hormones that normally prompt critical developmental processes and physiologic functions, including sexual behavior and reproduction. Because of this sensitivity, some chemicals—such as the industrial chemicals known as organochlorines—can interfere with endocrine function at extremely low environmental concentrations. Frogs are especially susceptible to developmental abnormalities because embryonic development takes place on the surface of the water, in open contact with any contaminants that may be there. Cricket frogs and other amphibians are therefore valuable sentinels of ecologic change.

The researchers examined museum specimens of cricket frogs that had been collected throughout Illinois for more than 150 years. This time span comprised five periods: a preorgano-chlorine period (1852–1929); a period of industrial growth and initial use of polychlorinated biphenyls (PCBs; 1930–1945); a period of rapidly increasing DDT use and further industrialization (1946–1959); a period of declining use and the eventual ban of DDT as well as the beginning of industrial pollution controls (1960–1979); and a period of substantial reduction of organochlorines in the environment (1980–2001). The team examined the specimens for evidence of “intersex” gonads—testes that produce egg cells or the presence of both a testicle and an ovary—which are known effects of endocrine disruptor exposure.

Once the most common amphibian in Illinois, cricket frogs have undergone a precipitous decline over the last 25 years to the point that they are now rarely seen in the north of the state. The research team’s findings suggest that increasing contamination with a suite of endocrine-disrupting organochlorine contaminants beginning in the 1930s likely contributed to the decline. Intersex frogs accounted for 1% of samples from 1852 to 1929, 7.5% of samples from 1930 to 1945, 17% of samples from 1946 to 1959, 10% of samples from 1960 to 1979, and 9% of samples from 1980 to 2001. The spatial analysis showed the effects to be the most pronounced in the urbanized, industrialized north of the state, around Chicago.

Furthermore, the researchers concluded that the greatest declines were seen in the areas with the most intersexuality: “The observed decline was evident following a period of sustained endocrine disruption, as indicated by a large increase in prevalence of intersex gonads and masculinization of the population,” they write. However, they add that they cannot conclude that the era of endocrine disruption in cricket frogs has come to an end, because the number of remaining cricket frogs is insufficient to permit sampling.

## Figures and Tables

**Figure:**
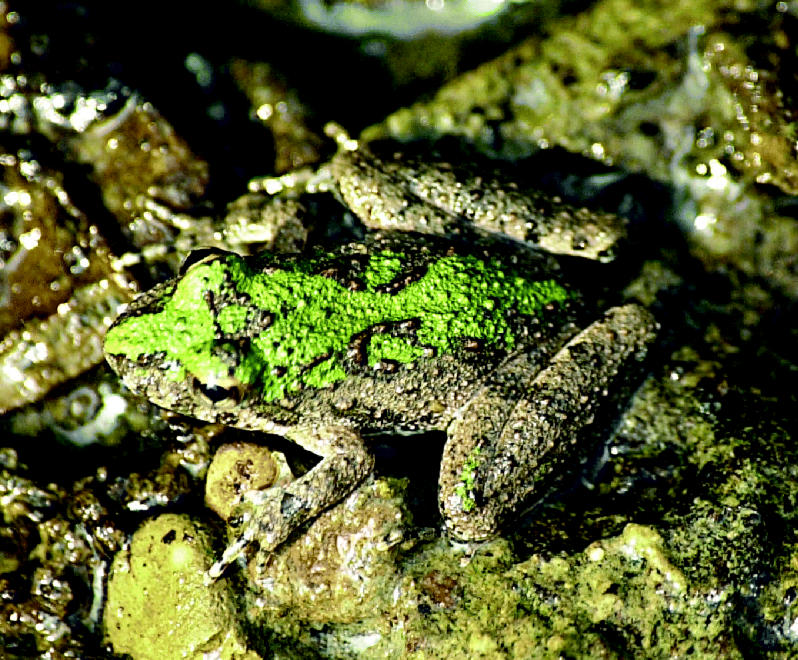
**Cricket frog swan song?** A new examination of historical data shows the decline of cricket frogs in Illinois correlates with organochlorine pesticide use.

